# Back to the future: Innovativeness in sound design for electrified vehicles

**DOI:** 10.1177/20416695251323786

**Published:** 2025-05-08

**Authors:** Mara Münder, Renzo Vitale, Claus-Christian Carbon

**Affiliations:** 1 Department of General Psychology and Methodology, University of Bamberg, Bamberg, Germany; 2 39346 BMW Group, Munich, Germany; 3 Bamberg Graduate School of Affective and Cognitive Sciences, Bamberg, Germany; 4 Research Group EPÆG (Ergonomics, Psychological Æsthetics, Gestalt), Bamberg, Germany

**Keywords:** sound design, innovation, electrified vehicle, perception, psychoacoustics, automotive, perceived quality, applied acoustics

## Abstract

With powertrain electrification, the character of a vehicle's soundscape significantly changes. Not only does it become quieter, but also the so far dominating combustion engine broadband noise, which shaped people's expectations, disappears. With the upcoming technology, we are empowered to create soundscapes with novel frequency spectra and sound design approaches. Vehicle soundscapes are dynamic by nature due to load and speed changes, and the driving situation. In this study, different active sound design concepts for electrified vehicles (EVs) have been assessed by *N* = 83 participants concerning their conveyed impressions of *Perceived Innovativeness*, *Liking*, and further relevant dimensions. The sound design concepts are varied in their general sound character and level of innovativeness and then presented in a listening laboratory. By employing the *Repeated Evaluation Technique* (RET) in the context of sound design research, we gather insightful data about dynamic effects in the perception of innovative acoustic textures in the context of active EV sound design. The findings of this study reveal dynamic effects in novelty perception and aesthetic preference, as well as insights into the semantics specific design characteristics convey. This can be helpful in shaping the character of a vehicle, making predictions about long-term appreciation of novel driving sound concepts, and assisting sound engineers and designers in creating innovative yet appreciated sound designs for EVs.

## How to cite this article

Münder, M., Vitale, R., & Carbon, C-C. (2025). Back to the Future: Innovativeness in Sound Design for Electrified Vehicles. *i-Perception*, 16(0), 1–24. https://doi.org/10.1177/20416695251323786

## Introduction

Accompanied by the electrification of vehicle powertrains, we currently face a drastic change in the noise profiles and acoustic quality of such vehicles. In the present paper, we focus on the impact of electrification on the interior soundscape of electrified vehicles (EVs). On the one hand, EVs are much quieter due to the overall reduced sound pressure level of emitted noise compared to conventional internal combustion engine vehicles (ICEVs) (Blickensdorff et al., 2019; Zeller, 2018). On the other hand, novel EV soundscapes with now unmasked disturbing noises (Blickensdorff et al., 2019; Eisele et al., 2019; Gavric, 2020), as well as EV-specific tonal components and higher frequency spectra (Blickensdorff et al., 2019; Devillers et al., 2020; Gavric, 2020) pose a challenge regarding the refinement of automotive acoustics (Allman-Ward et al., 2020; Gavric, 2020; Zeller, 2018). Noise, Vibration, and Harshness (NVH) criteria play a crucial role in customer satisfaction (Qatu, 2012; Qatu et al., 2009; Sinambari & Sentpali, 2020; Zeller, 2018), as they pose an immediate, multisensorial-perceivable entity, contributing to the overall quality impression of a vehicle (Münder & Carbon, 2022a). Beyond the naturally emitted noise by the electrified powertrain, active driving noise of EVs is a crucial NVH characteristic to be considered concomitant with the new technology in terms of sound quality (Allman-Ward et al., 2020; Genuit & Fiebig, 2014; Kleinjohann, 2020).

### Customer Expectations and Industry

Krishna (2021) revealed through a social media analysis that a vehicle's sound is one of the most significant assets through which customers build emotional connections and even might anthropomorphize it. Passionate car enthusiasts—so-called *petrol heads*—notoriously criticize the overall muted EV soundscapes as, for example, a “lackluster ‘appliance-like’” (p. 6) and “sterile” (p. 5) experience (Krishna, 2021). Public discourse on EV acoustics should be considered, as it determines customer expectations and acceptance (Fiebig & Schulte-Fortkamp, 2019; Genuit & Fiebig, 2014) toward the new technology in the transition phase. The association between the acoustic feedback of a vehicle and its perceived power and performance parameters has been strengthened over decades, making it difficult to resolve these links closely tied to the typical combustion engine soundscape in the customers’ minds (Cerrato, 2009; Clendinning, 2018). Borg (2014) even described electrified driving as contradicting “a lifetime of experiences and expectations about cars and sound” (p. 287). Contrary to the demands of an audible and familiar combustion-like sound character, common expectations for electrified driving seem to involve a quiet, almost gliding experience with yet driving-scenario-appropriate operational feedback (Genuit & Fiebig, 2014). Moreover, progressive-futuristic vehicle concepts from media such as sci-fi books, film, and television, which have been popularized in the 20th century, have to be considered in terms of today's customer expectations regarding futuristic sound textures (Clendinning, 2018). According to Fechner's Aesthetic Association Principle, associative factors, the individual's learning experience, and its cultural and epochal background, influence the aesthetic experience even more than direct factors, such as design properties (Ortlieb et al., 2020). Due to the scarce exposition and little experience most people have with electrified driving, it remains unclear if customers can even qualify what they expect of an electric vehicle's soundscape. Cerrato (2009) even assumed a transitional phase from familiar and less innovative sound concepts—resembling typical combustion engine noise—to increasingly innovative and daring novel approaches. Therefore, amid the transitional phase, it is up to the manufacturers to set a standard and define the driving acoustics in the era of EVs. As customers perceive pathbreaking products as more prototypical in their product domain (Carson et al., 2007), pioneering in active EV sound design can potentially be a competitive advantage. The interior of a car—including its interior soundscape—offers manifold design approaches to manufacturers (Leder & Carbon, 2005). According to Karlsson et al. (2003), it can be considered a user-driven product, making its design less restricted to technical constraints while allowing a stronger aesthetic and individualistic focus. Design facilitates the creation of emotional value and thereby potentially promotes customers’ loyalty and passion for the product, according to Noble and Kumar (2008), which they find especially important in light of current economic trends of relationship-focused customer strategies. Many manufacturers already recognize the importance of their EVs’ sound design, as some even team up with well-established composers and music producers (Kleinjohann, 2020), which underlines the particular importance of aesthetics and artistic facets in a car's design. Sound enhancement in EVs is discussed as a measure to re-establish familiar operational driving feedback, to create an emotionally charged and customizable driving experience, to shape distinct and product-unique characteristics or overall brand identity (Allman-Ward et al., 2020; Bodden & Belschner, 2016; Kleinjohann, 2020; Sottek et al., 2005; Streicher et al., 2021). Münder and Carbon (2022b) offer an overview of current research investigating different sound enhancement approaches to operational feedback, the semantics of sound concepts, and sound synthesis and enhancement strategies. Though some see a necessity in EV sound enhancement for various reasons (Allman-Ward et al., 2014; Kleinjohann, 2020), there is a lively debate in the field on the different possible approaches regarding its synthesis and design (Clendinning, 2018; Genuit & Fiebig, 2014). Within the target conflict of having the opportunity for quiet driving experiences versus requesting operational vehicle feedback as known (Fiebig & Schulte-Fortkamp, 2019), different strategies are discussed: conservative approaches to resemble ICEV-typical soundscapes and satisfy traditional expectations, the refinement of natural e-powertrain characteristics to preserve its authentic soundscape, or approaches with highly novel sound design concepts (Genuit, 2012; Genuit & Fiebig, 2014; Kleinjohann, 2020; Sinambari & Sentpali, 2020). Despite a large body of research, the question of the *best* strategy in EV sound enhancement seems to remain unclear, although a great variety of approaches seems feasible (Münder & Carbon, 2022b). Finding suitable driving sounds for EVs that highlight their outstanding vehicle dynamics, well-orchestrated with a unique, nearly silent driving experience, remains one of the main challenges in automotive acoustics. With these questions in mind, our study investigates what is considered a suitable degree of novelty in driving sounds for EVs and how stable preferences toward the different sound concepts are over repeated exposure.

There are also intra-individual factors that could influence the acceptance of or aversion to rather novel approaches in the design of a vehicle's interior soundscape among customers. One of these factors could be the customer's openness, one of the basic personality trait dimensions in the established five-factor models known as the *Big Five* (Rauthmann, 2017). Openness refers to how open a person tends to be toward new experiences. Soto and John (2017) adopted the label of open-mindedness instead of openness to distinguish between individual preferences regarding the range of cognitive, perceptual, and affective experiences from openness toward social experiences. In their *Big Five Inventory II* (BFI-II), they subsumed the facets of *intellectual curiosity*, *aesthetic sensitivity*, and *creative imagination* into the subscale of *Open-Mindedness* (Soto & John, 2017). A measure considering such tendencies specifically in the context of technology is the *Affinity for Technology Interaction* (ATI) scale by Franke et al. (2019), which can be helpful to characterize one's sample in technology interaction research, as it can have consequences on user experience and technology acceptance.

### Design Principles

As innovations often encounter customers’ resistance to adopting new products that have recently been introduced to the market, innovators should evaluate their concepts in early design stages to find out about the factors holding up product adoption that go beyond plain novelty (Heiskanen et al., 2007). According to Baha et al. (2012), radical innovations might lead to a dissociation of the novel product due to an imbalance in meaning: the novel character dominates and overshadows the existing product meaning, which is why consumers might not be able to relate anymore and reject the product's adoption. These assumptions align with a central design theorem of innovation established by industrial design pioneer Raymond Loewy: the MAYA-principle, standing for *Most Advanced Yet Acceptable* (Loewy, 1951). To avoid customer rejection, meaning gaps between the familiar combustion engine soundscape and the novel electrified soundscape need to be identified and bridged in terms of designing a suitable yet attractive driving sound (Baha et al., 2012). A possible way to introduce new meanings, stated by Baha et al. (2012), is to either combine existing meanings, or to build upon them as they are more relatable to customers in the specific product context. Hekkert et al. (2003) found a well-balanced combination of innovative and novel elements with typical and familiar attributes to be essential for aesthetic preference in their design studies.

### The Present Study

The main aim of the present study was to investigate dynamic effects in the perception of *Innovativeness* and *Liking*. Therefore, we applied the *Repeated Evaluation Technique* (RET) by Carbon and colleagues (Carbon, 2015; Carbon & Leder, 2005; Faerber et al., 2010; further details in the *Method* section), an evaluation method that, to our knowledge, has not been applied in the context of vehicle sound design before. By this novel methodological approach, we achieved a high degree of elaboration in the experimental listening lab setup and could compare the perception in those two dimensions over time to better predict a sound concept's future acceptance. The results of this study should guide automotive engineers in determining which sound design strategy to follow when shaping the sound characteristics of EVs.

Another study goal is to estimate an ideal degree of novelty in sound design approaches to identify an EV-driving sound with a high potential for long-term preference by evaluating different acoustic concepts. Therefore, we focus on sound concepts with varying levels of innovativeness and different sound characters and evaluate various dimensions such as *Liking*, *Perceived Innovativeness*, *Perceived Powerfulness*, *Perceived Sustainability*, *Perceived Pleasantness*, and *Perceived Emotionality*. The first two dimensions represent the main dimensions to investigate dynamic effects in matters of preference relating to novelty in sound concepts. The other four dimensions are chosen as they represent important characteristics of (novel) vehicles and help gather information about what certain sound textures transmit: the attributes of powerfulness and pleasantness are often discussed in research on interior sound design for EVs (e.g., Lennström et al., 2011; Ma et al., 2017; Swart et al., 2018; Swart & Bekker, 2019). Further, we were interested in the attributes of sustainability and emotionality, inspired by current design discussions in the applied field. Thereby, we are not only addressing aesthetic preference and novelty perception but also the semantic meaning the evaluated sound concepts convey. By considering these semantics in our investigation, we want to gain further insights into how powerful and strong (*Perceived Powerfulness*), how sustainable, green, and efficient (*Perceived Sustainability*), how pleasant, enjoyable, and comfortable (*Perceived Pleasantness*), and how affectively touching (*Perceived Emotionality*) the various concepts are perceived by our sample. The insights on conveyed semantic meaning can then be helpful in identifying the suitability of the different sound concepts for different vehicle types in terms of product characterization at a later stage in the design process.

In accordance with findings in the domain of visual aesthetics (see Carbon & Leder, 2005), we hypothesize that sound textures with higher degrees of novelty will be liked less in the course of a first assessment but will show increased preference ratings over further elaboration compared to less innovative sounds. For less innovative concepts, the preference ratings are suspected to remain relatively stable over time as the acoustic habits are already developed to process such sounds. Regarding the perceived novelty of the concepts, we expect that novelty aspects will wear off with increasing elaboration as they become more familiar over repeated exposure. Moreover, we presume that specific semantics are better liked (e.g., more pleasant sound textures) as they might be less annoying and stressful (Devillers et al., 2020; Gavric, 2020), perceived as more innovative (e.g., sound textures perceived as more sustainable), and possibly go hand in hand with each other (e.g., emotionality and powerfulness) as their semantic bonds have been strengthened over decades (Krishna, 2021), or interfere with one another (e.g., powerfulness and sustainability) as they are (at least currently) contradicting each other. To control for the factors potentially influencing the acceptance or reluctance of novel concepts, we further assess our sample's scores on the subscale of open-mindedness from the BFI-II (Soto & John, 2017) and on the ATI scale (Franke et al., 2019).

## Method

### Participants

We conducted an a priori power analysis for a repeated-measures analysis of variance (ANOVA), run in G*Power (Faul et al., 2007), aiming at a revealed medium effect size with a set statistical test power of 1‒β = 0.8 and an error probability of α = 0.05 to find the approximate sample size needed to test our main hypotheses.

The power analysis^1^ yielded a minimal required sample size of *N* ≥ 34. Two samples were recruited via email and notice board—one corporate sample at the BMW Group (*n* = 46) and one student sample at the University of Bamberg (*n* = 37; total sample size *N* = 83), to increase the diversity of our sample. Participating was not restricted except to normal hearing ability. We compensated participation only for students in the academic sample. The study was conducted according to the ethical principles expressed by the Declaration of Helsinki, the German Professional Psychologists (BDP), and the German Psychological Society (DGPs). Furthermore, the experimental plan was approved by the local ethics committee of the University of Bamberg. Prior to the experimental testing, participants were informed about the study's rationale without providing specific details on the employed stimuli. They all gave written consent to participate and were informed about their rights to withdraw from the study at any point in time without the need to state reasons. See Table 1 for more details on the samples.

**Table 1.** Demographic Sample Description.

	Overall	Corporate	Student
	*N*	*n*	*n*
Sample size	83	46 (55%^a^)	37 (45%^a^)
Gender			
Female	38 (46%^a^)	15 (33%^b^)	23 (62%^b^)
Male	45 (54%^a^)	31 (67%^b^)	14 (38%^b^)

	*M*	*SD*	*M*	*SD*	*M*	*SD*
Age (years)	30.1	9.0	33.2	10.3	26.1	4.8
Driving experience (years)	13	9	16	10	9	5

*Note.* Driving experience was defined through years of having a valid driving license; to avoid pseudo-accuracy, decimals were rounded for this variable.

a Percentage referring to overall sample size.

b Percentage referring to given sub-sample.

In total, 17% (*n* = 14) of sound design experts directly working on topics regarding automotive sound design participated in the study. Roughly a quarter of the sample (*n* = 20, i.e., 24%) has never driven an EV before—in the corporate sample, only 9% never drove electrically before, but in the student sample, it is the case for 43%. We further asked the participants for an estimate of how many hours per week they use vehicles with different powertrain systems: on average, our participants drove conventional cars with ICEVs approximately 3.5 hrs/week, followed by battery electric vehicles with 1.23 hrs/week, hybrid electric vehicles with 0.64 hrs/week, hydrogen cars 0.04 hrs/week and CNGG/LPG cars with 0.18 hrs/week. Additionally, we assessed the *Open-Mindedness* subscale for openness from the BFI II (Soto & John, 2017) and its three subscales, as well as the ATI scale (Franke et al., 2019), to further characterize our sample and control for biases between the samples. The results of the sample comparison are shown in Table 2.

**Table 2.** Sample Description in Openness (BFI-II) and Affinity for Technology (ATI).

Scale	Corporate Sample		Student Sample		Sample Comparison		
	*M*	*SD*	*M*	*SD*	*t(81)*	*p*	*d*
BFI-II Openness (total score)	46.85	6.20	46.11	5.49	0.57	.571	0.13
BFI-II Subscale Aesthetic Sensitivity	14.93	3.50	15.97	3.12	−1.41	.163	0.31
BFI-II Subscale Intellectual Curiosity	16.35	2.04	15.81	2.38	1.11	.271	0.24
BFI-II Subscale Creative Imagination	**15** **.** **57**	**2** **.** **52**	**14** **.** **32**	**2** **.** **46**	**2** **.** **25**	**.** 027*	**0** **.** **50**
ATI score	**4** **.** **71**	**0** **.** **82**	**3** **.** **72**	**1** **.** **01**	**4** **.** **95**	<.001***	**1** **.** **00**

*Note*. Results of *t*-test comparisons in personality variables. Significance levels: *p < .05; ***p < .001. The effect size magnitude (Cohen's) *d*, according to Cohen (1988), is defined as follows: *d*∼0.2 as a small, *d*∼0.5 as a medium, and *d*∼0.8 as a large effect.

Regarding openness, no significant differences were found between the two samples except on the subscale for *Creative Imagination*, as revealed by an independent *t*-test (*t*(81) = 2.25, *p* = .027*). The participants in the corporate sample (*M* = 15.57, *SD* = 2.52) reportedly score higher than the student sample (*M* = 14.32, *SD* = 2.46). The coefficient Cohen's *d* indicates a medium-sized effect (*d* = 0.50). When comparing the two samples in their scores on the ATI scale (Franke et al., 2019) through an independent *t*-test, the corporate sample (*M* = 4.71, *SD* = 0.82) shows significantly higher scores (*t*(81) = 4.95, *p* < .001***) with a large effect of *d* = 1.00 (see Table 2). When conducting *t*-tests, the assumptions regarding normality and homogeneity were met. Though the samples show slight differences in the variables mentioned above, no significant differences were found between the samples regarding the sound evaluation ratings in this study. Therefore, further analyses in this work were made with the overall sample.

### Apparatus

The study's centerpieces are the selected stimuli, all supposed to represent the acceleration of an EV. Due to proprietary reasons the authors cannot provide the employed stimuli to the public. However, we will do our best to describe the utilized material in the following passage. For the composition of the sound concepts, we focused on two variation parameters: the level of *Innovativeness* and the *Sound Character*. The level of *Innovativeness* represents a variation of more familiar concepts toward highly novel sound concepts. As there is no standard for innovativeness in sound design, we aimed for a wide variety of concepts without drifting off the actual context or having noncomparable concepts that are too far apart. Therefore, we utilized relatable and well-known noise spectra from familiar driving noise of ICEVs in the conventional category. For the progressive category, we used familiar elements in combination with novel acoustic features, for example, the combination of combustion engine-like pedal application in the vehicle's acceleration process with higher frequency spectra and tonal components or floating acoustic accompaniment throughout the entire acoustic scene. The novelty content then peaked in the futuristic category, where we employed musical sound components, dissociative running soundtracks, and abstract sound elements that have not been applied to standard production vehicles so far. Less innovative concepts were therefore assigned to the conventional category, novel sound elements and spherically floating soundscapes composed the progressive level, which was then developed further in the futuristic category in terms of even more spherically floating, cloudier, and enfolding soundscapes with unprecedented sound elements in the context of vehicle acoustics. This way, the familiarity with the sound concepts decreases with progressing *Innovativeness*. At the same time, the three levels of *Innovativeness* increasingly consider novel aspects in the two different *Sound Character* domains. For the variation in the *Sound Character*, determining the general, underlying sound character of the sound texture, we used either artistically or technically oriented sound elements. While musical elements guided the artistic concepts to create novel soundscapes, the technical concepts were oriented toward familiar acceleration mapping and mechanical concepts, resembling authentic machinery soundscapes.

From a wide range of sound concepts and drafted ideas gathered throughout different design processes within the BMW Group's acoustic and design department, the authors selected 15 sound concepts in the first step. Secondly, each author assigned the different concepts to the different cells within the variation matrix by themselves. Next, the authors brought together their categorizations of the sound concepts and discussed which stimuli represent each category best. After several internal review loops and careful consideration among the authors of this work, the sound material was narrowed down to 10 stimuli in total—all composed and produced by the composer and sound designer Renzo Vitale. The number of stimuli, all ranging between 14 and 25 s, was selected considering the experiment's duration and cognitive capacities of the study participants. As the conventional category appeared to be the most unambiguous due to its high degrees of familiarity, we decided to include only one stimulus to represent each of the *Sound Character*×*Innovativeness* combinations. The variation matrix is shown in Table 3: our final preset of stimuli consisted of 10 sound concepts in total, two for the conventional *Innovativeness Level* and four stimuli, each representing the progressive and the futuristic level. The stimuli were evenly distributed in the two *Sound Character* categories.

**Table 3.** Stimulus Variation Matrix.

Variation Matrix (Innovativeness × Sound Character)		Innovativeness		
		Conventional	Progressive	Futuristic
Sound character	Artistic	*Stim05*	*Stim06*	*Stim02*
			*Stim07*	*Stim03*
	Technical	*Stim10*	*Stim01*	*Stim04*
			*Stim08*	*Stim09*

*Note.* Stimulus matrix of the variation in *Innovativeness* and *Sound Character*.

To evaluate the sound concepts, we applied the RET developed by Carbon and colleagues (Carbon, 2015; Carbon & Leder, 2005; Faerber et al., 2010). Participants evaluated the aesthetics of the sound concepts regarding the two main dimensions, *Perceived Innovativeness* and *Liking*, as well as in the four semantic dimensions of *Perceived Sustainability*, *Perceived Powerfulness*, *Perceived Emotionality*, and *Perceived Pleasantness*, and finally on the two main dimensions again, a second time to gather data on potential dynamic perceptual changes due to the repeated exposure. As Carbon and Leder (2005) demonstrated that the familiarization phase provides a sufficient elaboration of the stimulus material, we gathered semantic data from the whole sample during the familiarization phase in terms of a within-participants experimental design. The study was self-programmed in Python and the acoustic stimuli were presented via Beyerdynamic^®^ DT 770 headphones while the scales were displayed on a computer screen. The playback volume for the stimuli was set to the same level for all participants after determining a suitable loudness among a small expert sample (*n* = 5) throughout the entire range of stimuli.

### Procedure

Each participant evaluated the sound concepts in a single evaluation session, lasting approximately one hour. Prior to the sound concept evaluation, the participants were each shown the same collage of nine different futuristic concept car interiors by various manufacturers (logos were blurred) for a total of 25 seconds (not skippable) to give them additional context for the following acoustic evaluation. All instructions were shown on a screen so the participants could go through the experiment at their own pace and take listening breaks between the test blocks whenever needed. The participants were encouraged to close their eyes while listening to foster the imagery of the EV context and acceleration process. In the first test block, the two main dimensions, *Perceived Innovativeness* and *Liking*, were evaluated, followed by the familiarization phase test block with the evaluation of the four semantic dimensions. The last test block consisted of the second evaluation of the two main dimensions. For each dimension, the stimuli were presented in randomized order, while the dimensions themselves in each test block were randomized in order as well. All evaluations were conducted on a seven-point Likert scale, where only the extrema were labeled, ranging from *1* (*not at all*) to *7* (*very much*). The questioned item was always visualized above the scale. For the preference dimension of *Liking*, we asked: “*How do you like the sound?*”, for the other dimensions, the question pattern stayed the same: “How *dimension* does this sound?” (*innovative, *powerful, *sustainable, *emotional, *pleasant). All words within the items referring to the respective dimension were highlighted in bold and blue-colored letters. After the experiment, the participants were asked for qualitative feedback on the study and the different sound concepts, which the study manager documented.

### Statistical Analysis

The statistical analysis was conducted through the open-source software R with the latest version available (version 4.4.1). Through the process of the analyses, we decided to switch from generalized linear models to linear mixed models (LMMs). The used models with their fixed and random effects are further described in the *Results* section when reported.

## Results

### Associations Between Preference, Novelty, and Semantic Dimensions

**Figure 1.**
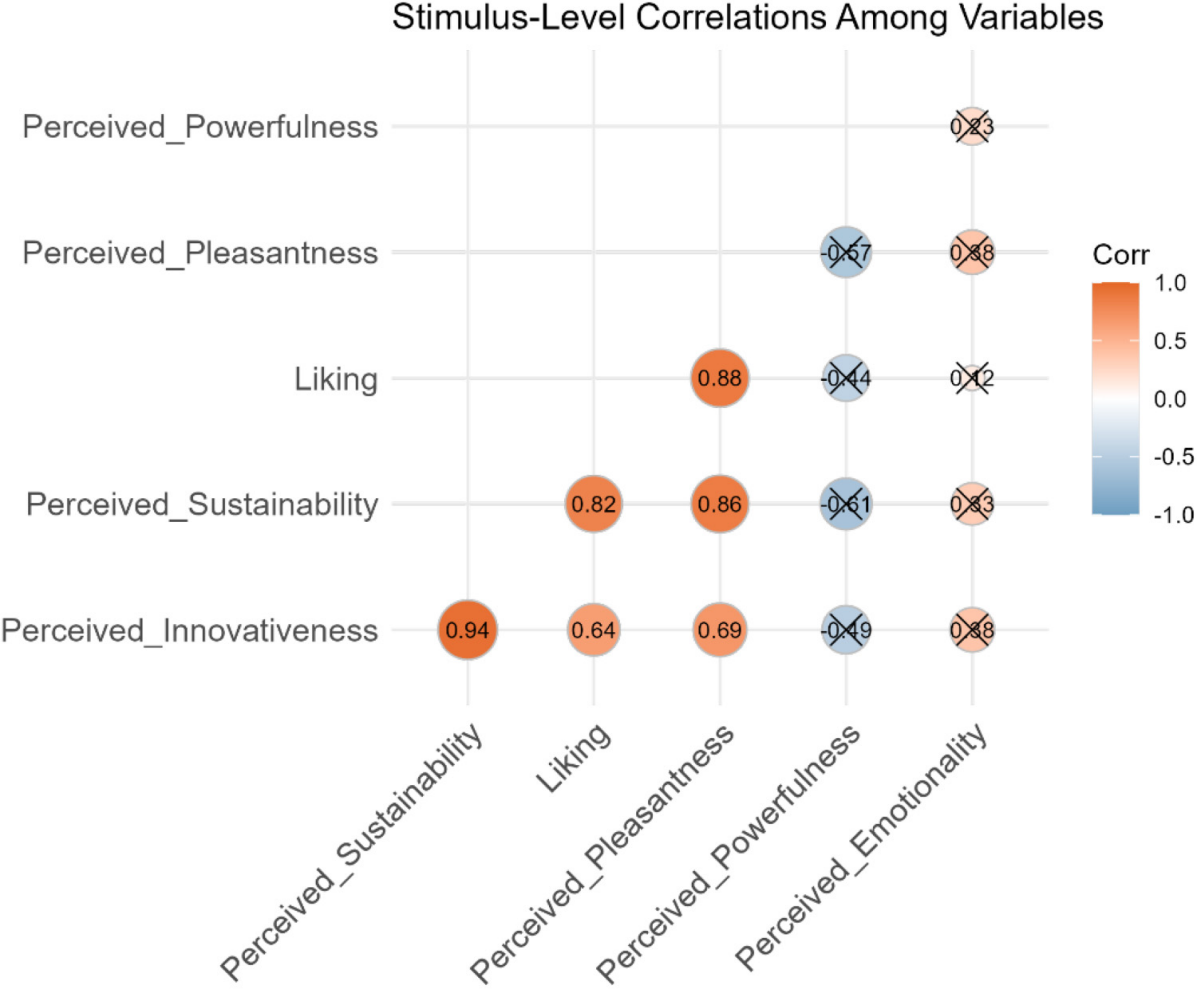
Correlation matrix in *T1*.

*Note.* Graphical depiction of the Pearson's correlations amongst the evaluated dimensions in *T1*. The correlations reflect the relationships among the variables as explained by the stimuli, independent of participant-level effects. The color indicates the valence of the correlation, the size of the circle its magnitude. Crossed coefficient circles are not significant.

We analyzed correlations between our tested main dimensions as well as between the main dimensions and the evaluated semantic dimensions. An overview of the associations between the evaluation dimensions is given in Table 4. *Liking* and *Perceived Innovativeness* are significantly positively correlated by *r* = .64 (*p* = .047*) at time point *T1* and by *r* = .73 (*p* = .015*) at *T2*. Therefore, the correlation seems to intensify over time from *T1* to *T2* as the correlation coefficient increases. The semantic dimensions *Perceived Pleasantness* (*r* = .88, *p* = .001**), which is consistent with our previous assumption (see *The Present Study*), and *Perceived Sustainability* (*r* = .82, *p* = .004**) are also significantly positively correlated with the dimension of *Liking*. The strongest correlation for the dimension of *Perceived Innovativeness* is significantly positive with *Perceived Sustainability* (*r* = .94, *p* < .001***), which is consistent with our previous assumption (see *The Present Study*), followed by a strong association with *Perceived Pleasantness* (*r* = .69, *p* = .028*). For *Perceived Powerfulness* and *Perceived Emotionality* no significant correlations are found. These findings support our hypothesis that specific semantics are associated with our main variables—such as pleasant sounds being liked better and sounds conveying sustainability being associated with high degrees of novelty. Among the semantic dimensions, only *Perceived Sustainability* and *Perceived Pleasantness* are significantly associated with one another (*r* = .86, *p* = .001**). Our study's results could not confirm a *significant* negative correlation between *Perceived Powerfulness* and *Perceived Sustainability* as stated in our assumptions (see *The Present Study*), but a trend toward a negative correlation was quite obvious (*r* = −.61, *p* = .062). Nevertheless, we could not confirm a significant association between *Perceived Powerfulness* and *Perceived Emotionality* (*r* = .23, *p* = .520). Figure 1 graphically depicts the correlations among the variables.

**Table 4.** Correlations of Evaluation Dimensions.

Pearson's *R*	*Liking*	*Perceived Innovativeness*	*Perceived Powerfulness*	*Perceived Sustainability*	*Perceived Emotionality*	*Perceived Pleasantness*
*T1*						
*Liking*		.64*	−.44	.82**	.12	.88**
*p*-value		.**047**	.206	.**004**	.735	.**001**
*df*		**8**	8	**8**	8	**8**
*Perceived innovativeness*	.64*		−.49	.94***	.38	.69*
*p*-value	.**047**		.149	**<.001**	.274	.**028**
*df*	**8**		8	**8**	8	**8**
*Perceived powerfulness*	−.44	−.49		−.61	.23	−.57
*p*-value	.206	.149		.062	.520	.087
*df*	8	8		8	8	8
*Perceived sustainability*	.82**	.94***	−.61		.33	.86**
*p*-value	.**004**	**<.001**	.062		.350	.**001**
*df*	**8**	**8**	8		8	**8**
*Perceived emotionality*	.12	.38	.23	.33		.38
*p*-value	.735	.274	.520	.350		.282
*df*	8	8	8	8		8
*Perceived Pleasantness*	.88**	.69*	−.57	.86**	.38	
*p*-value	.**001**	.**028**	.087	.**001**	.282	
*df*	**8**	**8**	8	**8**	8	
*T2*						
*Liking*		.73*				
*p*-value		.**015**				
*df*		**8**				
*Perceived innovativeness*	.73*					
*p*-value	.**015**					
*df*	**8**					

*Note.* Cross table for Pearson's correlations between the evaluation dimensions and semantic dimensions. The correlations reflect the relationships among the variables as explained by the stimuli, independent of participant-level effects. Note that the second measurement time point *T2* was only assessed for the two main dimensions *Liking* and *Perceived Innovativeness*. Significant results are indicated by boldface type. Significance levels: *p < .05; **p < .01; ***p < .001. Interpretation of correlation coefficients following Cohen (1988) as follows: *r* ≥ .10 and *r* < .30 as a weak correlation, *r* ≥ .30 and *r* < .50 as a moderate correlation, and *r* ≥ .50 as a strong correlation.

### Data Distribution for *Liking* and *Perceived Innovativeness*

**Figure 2.**
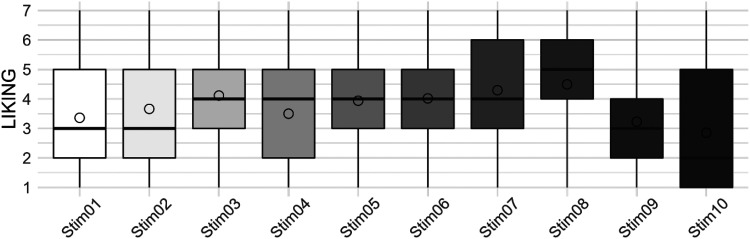
*Liking* ratings across stimuli.

*Note.* Ratings for each stimulus in the dimension of *Liking*. The boxplots show the rating distribution for each stimulus; the black bar in the box represents the median, while the circles represent the mean ratings.

For the evaluation of the *Liking* dimension, the participants used the full range of the scale (see Figure 2), demonstrating a high degree of interindividuality in the preference for one or the other sound concept. The stimulus *Stim08* was liked the most, followed by *Stim07* and *Stim03*.

We analyzed the empirical innovativeness that the stimuli conveyed, as we could not resort to a defined standard for innovativeness in EV sound design and settled for a wide range of stimuli through our expertise. Table 5 shows the statistical values in the dimension of *Perceived Innovativeness* for our 10 stimuli.

**Table 5.** Statistical values of *Perceived Innovativeness*.

Stimulus	*M*	*SD*	*MD*	Stimulus	*M*	*SD*	*MD*
*Stim01*	3.07	1.37	3.00	*Stim06*	4.84	1.30	5.00
*Stim02*	5.63	1.18	6.00	*Stim07*	5.59	1.27	6.00
*Stim03*	5.02	1.24	5.00	*Stim08*	4.63	1.79	5.00
*Stim04*	5.15	1.26	5.00	*Stim09*	4.27	1.41	4.00
*Stim05*	4.84	1.19	5.00	*Stim10*	1.44	0.93	1.00

*Note.* Statistical values for the dimension of *Perceived Innovativeness*. For each stimulus, both evaluation time points (test block one and test block three) are considered.

**Figure 3.**
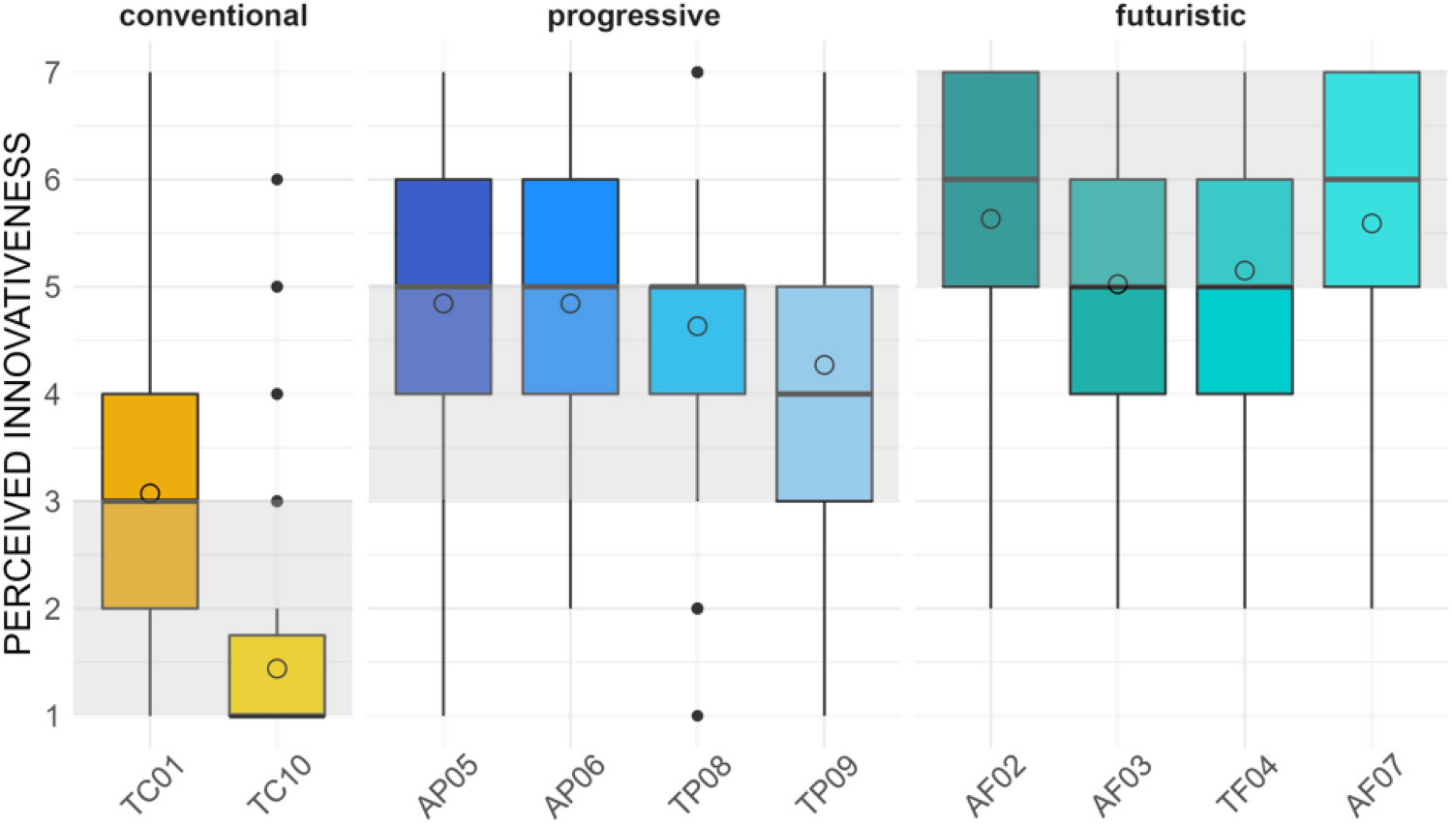
Ratings for *Perceived Innovativeness* across stimuli.

*Note.* Empirical data distribution for each stimulus in the dimension of *Perceived Innovativeness* grouped by *Innovativeness Level*. The segmentation of the scale into the three different *Innovativeness Levels* is indicated by the highlighted shadowed areas. The boxplots show the rating distribution; the median is indicated by the thick black line across the box, the whiskers of the boxes extend to the most extreme data points, while the black dots represent outliers. The hollow black circles indicate the mean value (both evaluation time points included).

According to the empirical values, our preset shows a good fit regarding the different innovativeness levels overall. If we split up our 7-point Likert scale into three segments—corresponding to three innovativeness levels—we have a cut-off and maximum value for the *conventional* level at a mean of 3.00, for the *progressive* level at a mean of 5.00 and for the *futuristic* level a mean that needs to be higher than 5.00. The ascription of the different stimuli to the segmented innovativeness levels by their empirical *Perceived Innovativeness* is depicted in Figure 3.

To simplify the attribution to the variation matrix, the stimuli are, from now on, encoded more descriptively by indicating their category of *Sound Character* by the first letter (“*A*” = *artistic*, “*T*” = *technical*), the *Innovativeness Level* by a second letter (“*C*” = *conventional*, “*P*” = *progressive*, “*F*” = *futuristic*), and a serial number from the conception phase. This results in *TC01* (*Stim01*; slightly tearing the maximum value but considered as conventional to have a better stimulus distribution amongst the innovativeness groups) and *TC10 (Stim10)* to be ascribed to the *conventional* level. None of the artistic concepts are ascribed to the *conventional* level, considering their empirical values. *AP05 (Stim05)*, *AP06 (stim06)*, *TP08 (Stim08)*, and *TP09 (Stim09)* define the *progressive* level, and *AF02 (Stim02)*, *AF03 (Stim03)*, *TF04 (Stim04)*, and *AF07 (Stim07)* are ascribed to the *futuristic* level. Regarding the rating distribution in the dimension of *Perceived Innovativeness* only for *AP05*, *TC01*, and *TP09*, the full range of the scale is used. At the same time, the participants seem to agree more with the innovativeness estimation for *TC10* and *TP08*.

To compare the effects of different variables on our participants’ ratings, we statistically tested the effect of different degrees of novelty in a sound concept (conventional, progressive, futuristic) and a variation of the base sound character (technical, artistic) on the ratings, as well as the effect of repeated evaluation (time point) on the perceived impression of novelty and the preference for a sound concept (the two main dimensions: *Liking*, *Perceived Innovativeness*) by means of LMMs. We first defined a null model (*M0*) considering factors for which we only considered the random effects of each participant (*ID*) on the ratings. In our model *M1* we added variables, for which we had no specific hypothesis in mind: *age* (of the participant), *gender* (of the participant), *ATI* (affinity toward technology measured by the numeric score from the *ATI*), and *Open* (numeric score from the *BFI-II* regarding the participant's open-mindedness toward new experiences) as fixed effects and the *ID* (participant) as random effect. Proceeding from this model *M1* we added further fixed effects we suspected to influence our participant's ratings to our *M2*: *InnoLev* (the novelty variation within our sound concepts by the *Innovativeness Level*: conventional, progressive, futuristic), *SoundChar* (the basic *Sound Character* of our sound concepts technical, artistic), *time* (indicating if the evaluation was repeated, i.e., test phase 1 and 2, so *T1* and *T2* in the two main dimensions of *Liking* and *Perceived Innovativeness*), *profession* (sound design, acoustics, innovations, other), and the respective *dimension*. For the model *M3* we then added the interaction of the variation variables of our stimuli (*SoundChar* and *InnoLev*) within each evaluation dimension, following the idea that a specific combination of the variation parameters creates specific impressions that are more suitable for specific dimensions than others. The different models are described in Table 6.

**Table 6.** Models.

Model	*Npar*	*AIC*	−2LL	*df*	*X*²	*p*
*M0*: 1 + (1|ID)	7	25,664	−12,825			
*M1*: 1 + age + gender + ATI + Open + (1|ID)	**8**	**24,695**	**−12,340**	**1**	**971** **.** **18**	<.001***
*M2*: 1 + age + gender + ATI + Open + profession + dimension + InnoLev + SoundChar + time + (1|ID)	**20**	**24,502**	**−12,231**	**12**	**217** **.** **69**	<.001***
*M3*: 1 + age + gender + ATI + Open + profession + dimension + InnoLev*SoundChar + time + (1|ID)	21	24,503	−12,231	1	0.14	0.711

*Note*. *Npar* = number of model's parameters; *AIC* = Akaike information criterion, an estimator of prediction error; *−2LL* = likelihood ratio; *df* = degrees of freedom; *p* = *p*-value of the regarding χ^2^-test. Significant results are indicated by boldface type. Comparison of the models amongst each other (always comparing the present model with the preceding one, e.g., the line for *M2* indicates the comparison between *M1* and *M2*). Significance levels: *p < .05; **p < .01; ***p < .001.

When comparing our models with the *anova()* function, further demographic variables (*M1*) and further fixed effects (*M2*) explain significantly more variance compared to *M0*. The inclusion of the interaction effect between our variation parameters of novelty in the sound concept (*InnoLev*) and its basic sound character (*SoundChar*) does not significantly explain more variance in our model (comparison of *M2* and *M3*). Therefore, we proceeded with the evaluation of *M2* within each dimension (*Liking*, *Perceived Innovativeness*, *Perceived Sustainability*, *Perceived Powerfulness*, *Perceived Emotionality*, *Perceived Pleasantness*), displaying the fixed effects for each variable in Table 7.

**Table 7.** Detailed Results of Model *M2*.

	Liking			Perceived Innovativeness		
Predictors	Estimates	*p*	*df*	Estimates	*p*	*df*
(Intercept)	**3**.96***	**<.001**	**79.53**	**3**.31***	**<.001**	**78.25**
age	**−0**.02**	**.002**	**75**	**−0**.01*	.**045**	**75**
ATI score	0.01	.873	75	−0.01	.885	75
Openness score	0.00	.684	75	0.00	.968	75
gender_female	*Reference*			*Reference*		
gender_male	0.10	.506	75	−0.04	.784	75
profession_acoustics	*Reference*			*Reference*		
profession_sounddesign	−0.36	.118	75	−0.36	.091	75
profession_other	−0.32	.129	75	−0.11	.591	75
profession_student	**−0**.41*	**.047**	**75**	−0.17	.386	75
InnoLev_conventional	*Reference*			*Reference*		
InnoLev_progressive	**0**.67***	**<.001**	**1573**	**2**.22***	**<.001**	**1573**
InnoLev_futuristic	**0**.57***	**<.001**	**1573**	**2**.84***	**<.001**	**1573**
SoundChar_artistic	*Reference*			*Reference*		
SoundChar_technical	**−0**.29**	**.001**	**1573**	**−0**.34***	**<.001**	**1573**
Time_T1	*Reference*			*Reference*		
Time_T2	**0**.23**	**.003**	**1573**	**−0**.21***	**<.001**	**1573**

	Perceived Powerfulness			Perceived Sustainability		
Predictors	Estimates	p	df	Estimates	p	df
(Intercept)	**4**.94***	**<.001**	**80.22**	**3**.87***	**<.001**	**77.99**
age	**−0**.02*	**.013**	**75**	−0.01	.161	75
ATI score	−0.06	.526	75	−0.06	.564	75
Openness score	0.01	.586	75	−0.00	.980	75
gender_female	*Reference*			*Reference*		
gender_male	−0.05	.784	75	−0.07	.730	75
profession_acoustics	*Reference*			*Reference*		
profession_sounddesign	−0.28	.303	75	−0.36	.237	75
profession_other	−0.38	.132	75	−0.35	.237	75
profession_student	**−0**.56*	**.023**	**75**	−0.31	.285	75
InnoLev_conventional	*Reference*			*Reference*		
InnoLev_progressive	−0.10	.509	744	**1**.75***	**<.001**	**744**
InnoLev_futuristic	0.02	.884	744	**2**.00***	**<.001**	**744**
SoundChar_artistic	*Reference*			*Reference*		
SoundChar_technical	**0**.63***	**<.001**	**744**	**−0**.63***	**<.001**	**744**

	Perceived Pleasantness			Perceived Emotionality		
Predictors	Estimates	p	df	Estimates	p	df
(Intercept)	**5**.04***	**<.001**	**79.30**	**3**.22***	**<.001**	**80.95**
age	−0.01	.346	75	0.00	.846	75
ATI score	−0.06	.576	75	0.05	.558	75
Openness score	−0.02	.283	75	0.02	.188	75
gender_female	*Reference*			*Reference*		
gender_male	−0.05	.787	75	−0.23	.178	75
profession_acoustics	*Reference*			*Reference*		
profession_sounddesign	−0.47	.125	75	**−0**.63*	.**022**	**75**
profession_other	−0.04	.889	75	−0.30	.241	75
profession_student	−0.14	.619	75	−0.23	.345	75
InnoLev_conventional	*Reference*			*Reference*		
InnoLev_progressive	**0**.84***	**<.001**	**744**	**0**.42*	.**011**	**744**
InnoLev_futuristic	**0**.84***	**<.001**	**744**	**0**.76***	**<.001**	**744**
SoundChar_artistic	*Reference*			*Reference*		
SoundChar_technical	**−0**.59***	**<.001**	**744**	**−0**.46***	**<.001**	**744**

*Note.* Fixed effects for different predictor variables in the six evaluation dimensions. The time variable is only included in the two main dimensions of *Liking* and *Perceived Innovativeness* as the other dimension variables were only evaluated at one time point. Significant results are indicated by boldface type. Significance levels: *p < .05; **p < .01; ***p < .001.

Throughout all dimensions the demographic variables seem to have no to little influence on the perception of the presented sound concepts. An exception seems to be the professional background regarding some of the rating dimensions. According to our results, students seem to have liked the sound concepts less overall (*b* = −0.41, *p* = .047*) and perceived them as significantly less powerful overall (*b* = −0.56, *p* = .023*) compared to the other professional groups. The group of sound designers within our sample there again perceived the sound concepts as less emotional overall (*b* = −0.63, *p* = .022*) compared to the other professional groups in the study. The variation of novelty (*Innovativeness Level*) within the sound concepts showed significant effects in various dimensions: the progressive (*b* = 0.67, *p* < .001***) and futuristic (*b* = 0.57, *p* < .001***) sound concepts seem to be better liked compared to the presented conventional sounds. Both innovativeness levels are also perceived as more innovative compared to the conventional level, with the futuristic level (*b* = 2.84, *p* < .001***) being perceived as more innovative than the progressive level (*b* = 2.22, *p* < .001***), which further supports the categorization of our variation matrix. Further, the sound concepts of the progressive and futuristic levels are perceived as significantly more sustainable (progressive: *b* = 1.57, *p* < .001***; futuristic: *b* = 2.00, *p* < .001***), pleasant (both levels: *b* = 0.84, *p* < .001***), and emotional (progressive: *b* = 0.42, *p* = .011*; futuristic: *b* = 0.76, *p* < .001***), while for the *Perceived Powerfulness* we could not find a significant effect of the variation within the three *Innovativeness Levels*. The variation of the general *Sound Character* of the stimulus shows significant effects throughout all evaluated dimensions: more technical sound concepts are overall less liked (*b* = −0.29, *p* = .001**) and perceived as less innovative (*b* = −0.34, *p* < .001***), less sustainable (*b* = −0.63, *p* < .001***), less pleasant (*b* = −0.59, *p* < .001***), and less emotional (*b* = −0.46, *p* < .001***), while being perceived as more powerful (*b* = 0.63, *p* < .001***) compared to the artistic sound concepts. Regarding the dynamic effects, which we tested for the main dimensions of *Liking* and *Perceived Innovativeness* through repeated evaluation, our results indicate that the sound concepts are better liked (*b* = 0.23, *p* = .003**) and perceived as less innovative (*b* = −0.21, *p* < .001***) over time.

### Analysis of Dynamic Effects in *Liking* and *Perceived Innovativeness*

**Figure 4.**
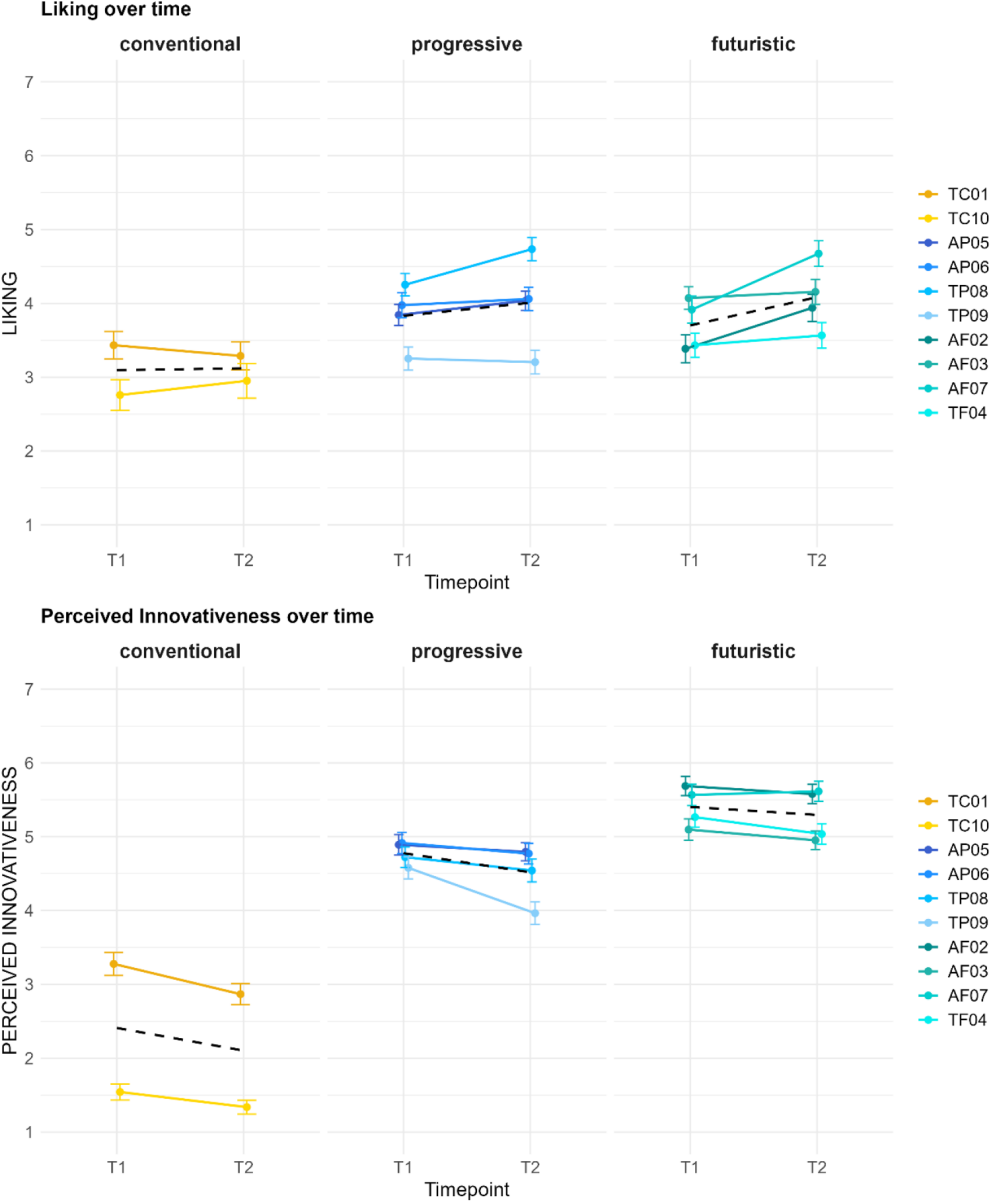
Dynamic effects in *Liking* and *Perceived Innovativeness*.

*Note. Liking* and *Perceived Innovativeness* ratings per stimulus over time for each *Innovativeness Level*. The overall trend is indicated by the black dashed line (no error bars) depicting the overall mean of all stimuli in the given dimension. The order of the items in the legends corresponds to their scores in *T2* to reduce complexity while reading. Error bars indicate ±1 standard deviation.

As we asked for a repeated evaluation of the dimensions of *Liking* and *Perceived Innovativeness*, we can compare the participants’ ratings over time. Figure 4 shows the ratings on the mentioned dimensions per stimulus in the first evaluation (*T1*) and the second evaluation after the extended elaboration of the sound concepts (*T2*).

To analyze the variances in the ratings over time, we considered the *time* (first or second evaluation time point) and the *stimulus* as fixed effects and the individual participants (*ID*) as random effect in a smaller model. In the resulting LMM, we found the sound concepts *AF03* (*b* = 0.64, *p* < .01**), *AP06* (*b* = 0.54, *p* < .05*), *AF07* (*b* = 0.48, *p* < .05*), and *TP08* (*b* = 0.82, *p* < .001***) to be significantly liked better, while *TC10* was significantly liked less (*b* = −0.67, *p* < .01**) overall. After extended elaboration (in *T2*), the sound concepts of *AF02* (*b* = 0.70, *p* < .05*) and *AF07* (*b* = 0.90, *p* < .01**) were even liked significantly better than in the first evaluation (*T1*). For the time points, the model does not find any significant dynamic effects, whereas when solely considering the time points as fixed effects, the *Liking* seems to increase over time (*T2*: *b* = 0.23, *p* < .01**). Though no significant dynamic effects for the individual stimuli were found in the dimension of *Perceived Innovativeness*, overall, it seems to wear off with time as a significant dynamic effect for *T2* (*b* = −0.41, *p* < .05*) is found within our model.

### Dynamic Effects for the Variation of *Sound Character* and *Innovativeness Level*

**Figure 5.**
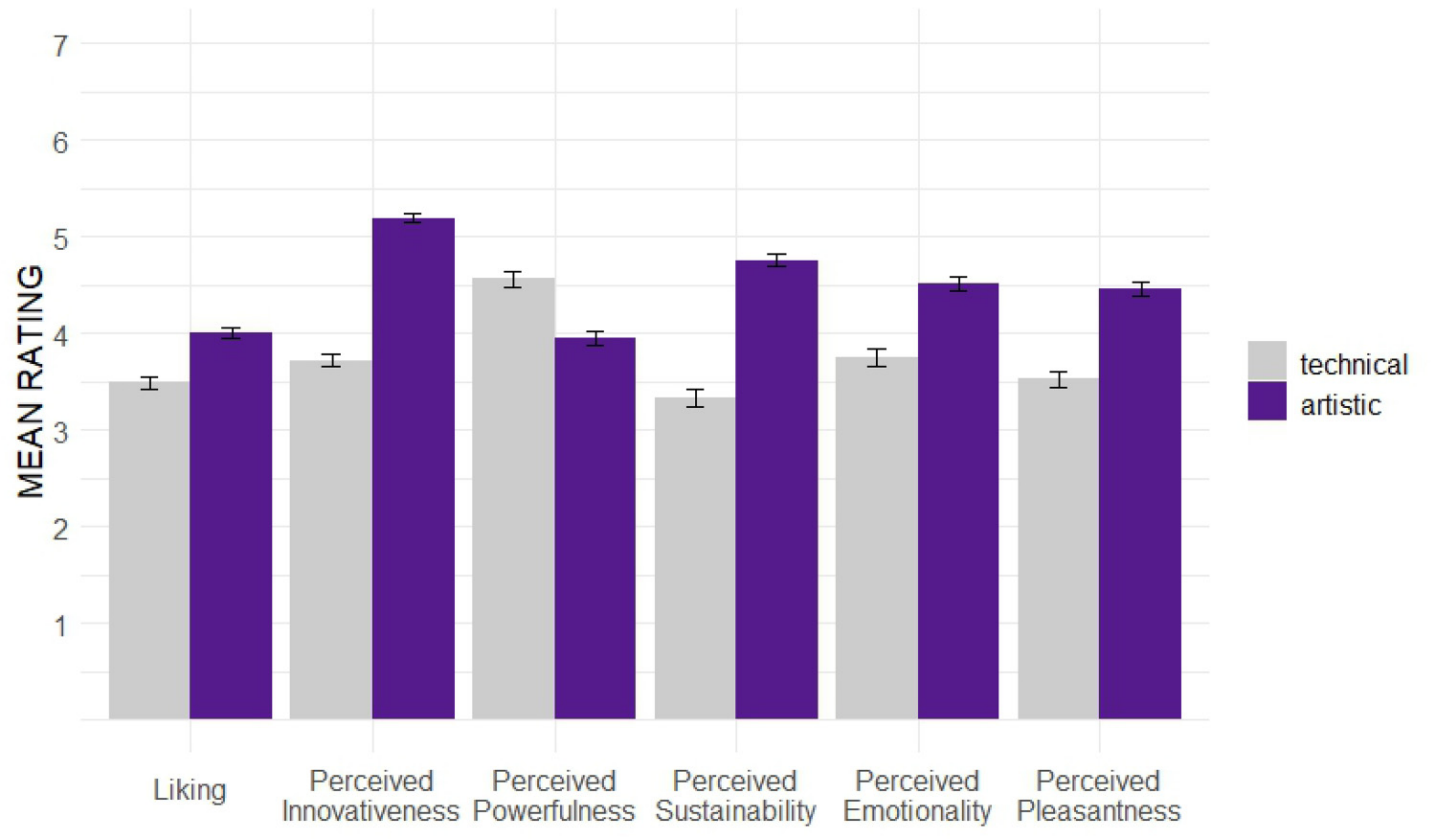
Mean ratings per *Sound Character* group.

*Note.* Mean ratings per evaluation dimension (both time points considered for the dimensions of *Liking* and *Perceived Innovativeness*) grouped by *Sound Character* (*technical* and *artistic*). Error bars indicate ±1 standard deviation.

The following section focuses on our research questions regarding the dynamic effects of aesthetic preference and novelty perception over time. At first, we will take a closer look at the underlying character of the sound textures, determined by the *Sound Character* groups. Figure 5 shows the overall mean ratings for all six evaluation dimensions per *technical* and *artistic* group.

**Figure 6.**
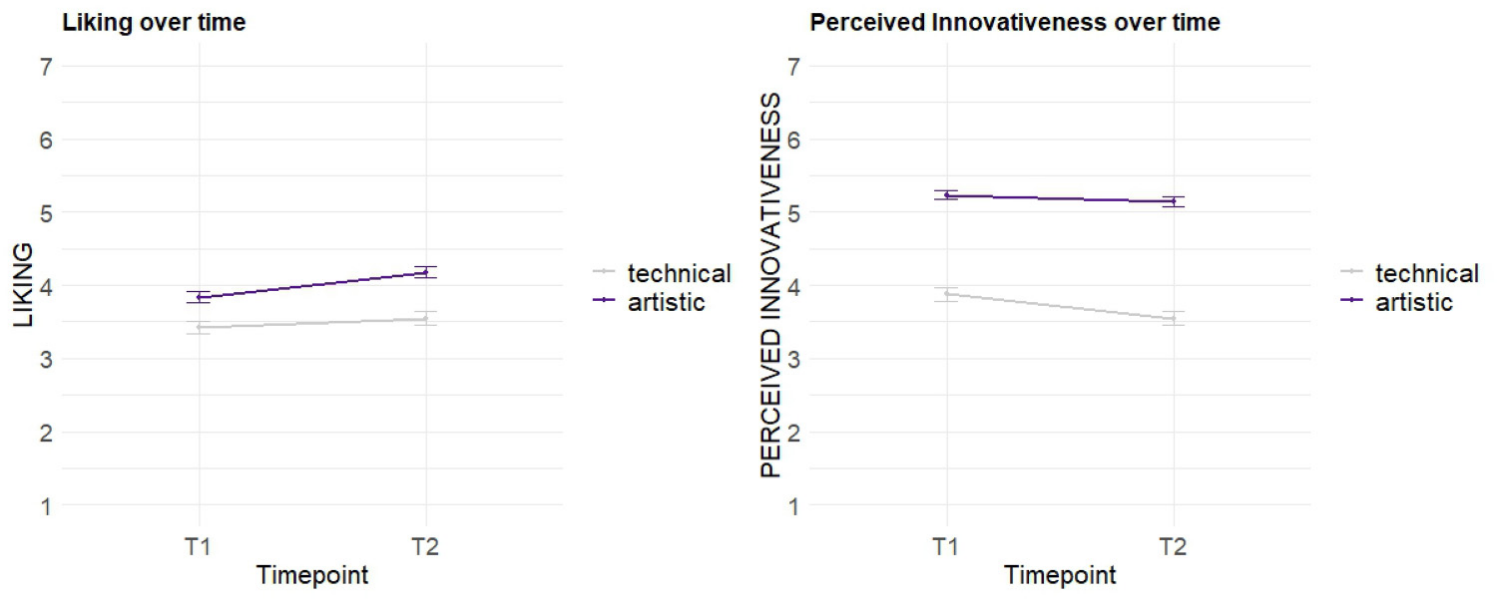
Dynamic effects per *Sound Character* group.

*Note.* Mean ratings of *Liking* and *Perceived Innovativeness* over time (first evaluation *T1* and second evaluation after extended elaboration *T2*) grouped by *Sound Character* (*technical* and *artistic*). Error bars indicate ±1 standard deviation.

The dynamic effects in the evaluation of the two main dimensions *Liking* and *Perceived Innovativeness* are depicted in Figure 6.

In an LMM, considering the time point of evaluation and the *Sound Character* as fixed effects and the individual participant as random intercept, the *Liking* overall significantly increases over time (*b* = 0.33, *p* < .01**). This is consistent with our hypothesis (see *The Present Study*) and indicates elaboration to play an important role in terms of preference evaluation. The *technical* sound concepts are found to be significantly less liked (*b* = −0.41, *p* < .001***) compared to the *artistic* sound concepts. No significant dynamic effects when comparing the two *Sound Character* groups were found. However, the graph shows a slightly more positive trend for the *artistic* sound concepts in *T2* than for the *technical* concepts. In the LMM for *Perceived Innovativeness*, we found a significant dynamic effect of *Perceived Innovativeness* decreasing with time (*b* = −0.33, *p* < .01**), supporting our hypothesis that perceived novelty in the sound concepts wears off over time. Moreover, the *artistic* sound concepts were perceived as significantly more innovative overall (*b* = 1.35, *p* < .001***) than the *technical* ones. Per *Sound Character* group, no significant dynamic effects were found, though the graph for the *technical* group shows a steeper decrease, whereas the novelty impression seems to be more stable over time for *artistic* concepts.

**Figure 7.**
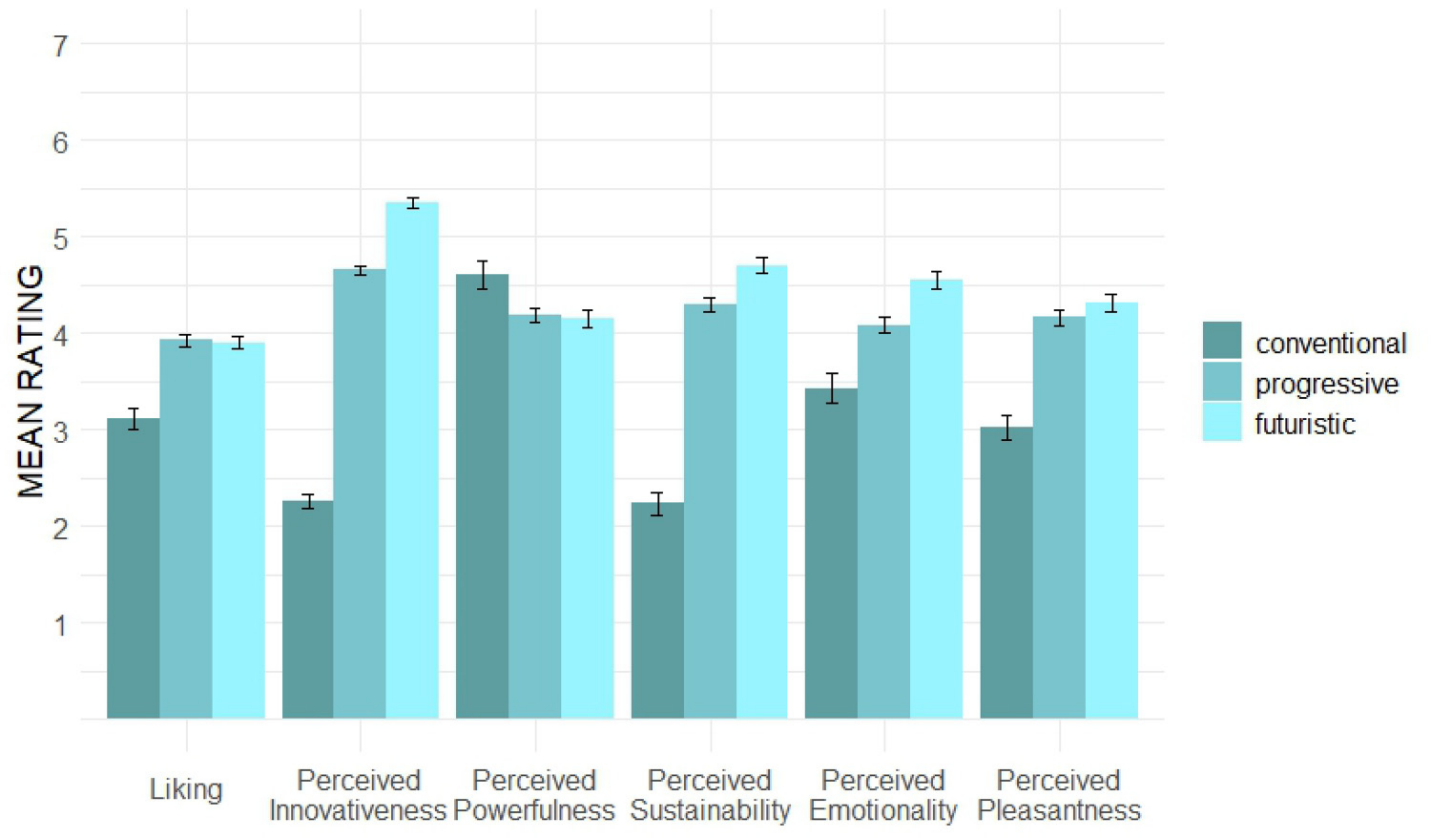
Mean ratings per *Innovativeness Level*.

*Note.* Mean ratings per evaluation dimension (both time points considered for *Liking* and *Perceived Innovativeness*) are grouped by *Innovativeness Level* (*conventional*, *progressive*, *futuristic*). Error bars indicate ±1 standard deviation.

After grouping the sound concepts according to their general sound character, we focused on the different *Innovativeness Levels*. The overall mean ratings for the three different levels in all six evaluation dimensions are shown in Figure 7.

In our LMM, we considered the *Innovativeness Level* as a fixed effect and the individual participant as random intercept for each dimension. The *progressive* (*b* = 0.81, *p* < .001***) and *futuristic* (*b* = 0.78, *p* < .001***) levels are significantly more liked than the *conventional* one. Regarding the *Perceived Innovativeness*, all levels are perceived as significantly innovative and in the expected order: the *futuristic* (*b* = 3.09, *p* < .001***) and *progressive* (*b* = 2.39, *p* < .001***) levels are perceived as significantly more innovative than the *conventional*. The concepts of the latter, nonetheless, seem to be perceived as more powerful, as the *futuristic* (*b* = −0.45, *p* < .01**) and *progressive* (*b* = −0.41, *p* < .01**) levels are perceived as significantly less powerful. Thereagainst, the two higher levels are perceived as significantly more sustainable (*futuristic*: *b* = 2.47, *p* < .001***; *progressive*: *b* = 2.06, *p* < .001***), as well as perceived as significantly more pleasant (*futuristic*: *b* = 1.29, *p* < .001***; *progressive*: *b* = 1.14, *p* < .001***) compared to the *conventional* level. Moreover, the *futuristic* level (*b* = 1.11, *p* < .001***), followed by the *progressive* level (*b* = 0.65, *p* < .001***), is perceived as significantly more emotional in comparison with the *conventional* level.

**Figure 8.**
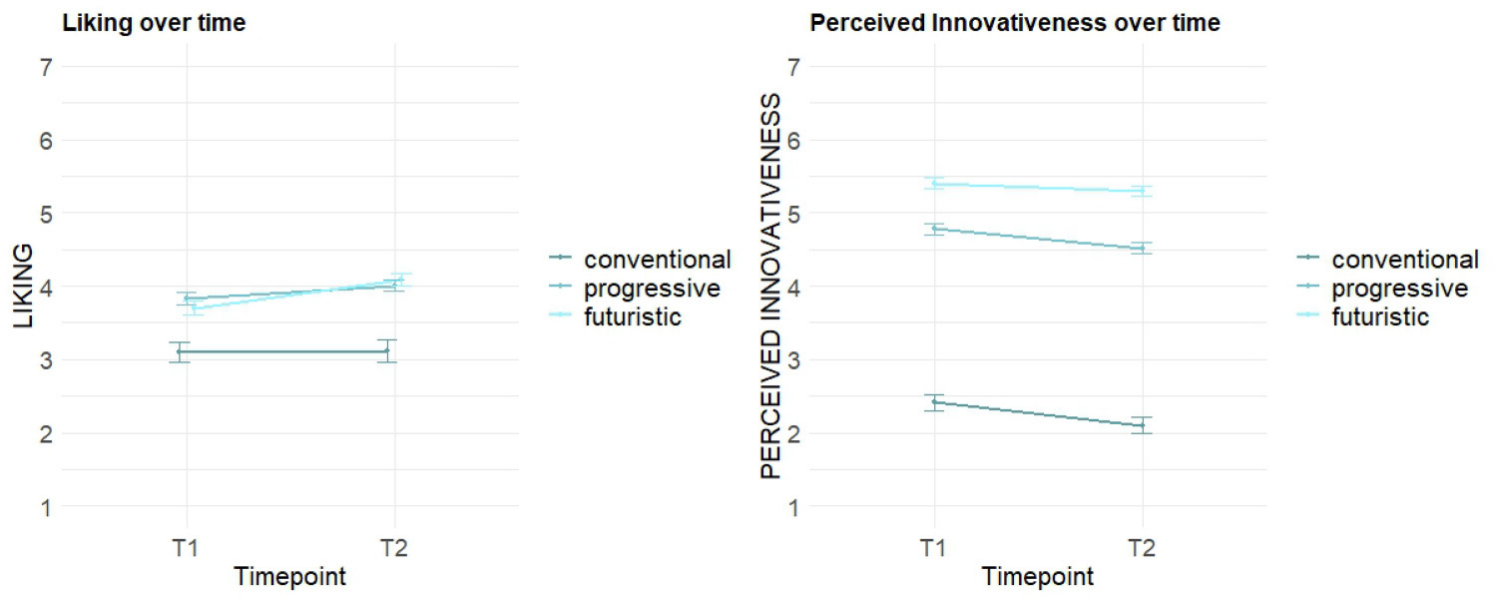
Dynamic effects per *Innovativeness Level*.

*Note.* Mean ratings in *Liking* and *Perceived Innovativeness* over time (first evaluation *T1* and second evaluation after extended elaboration *T2*) grouped by *Innovativeness Level* (*conventional*, *progressive*, *futuristic*). Error bars indicate ±1 standard deviation.

The dynamic effects in the evaluation of *Liking* and *Perceived Innovativeness* from the first and second evaluation is depicted in Figure 8. To analyze the time-dependent differences, we fitted an LMM with time points and *Innovativeness Level* as fixed effects and the individual as a random intercept. Sound concepts from the *futuristic* (*b* = 0.61, *p* < .001***) and *progressive* (*b* = 0.73, *p* < .001***) levels are significantly better liked compared to the *conventional*. For the *Perceived Innovativeness*, we applied a similar LMM, finding the *futuristic* (*b* = 2.99, *p* < .001***) and *progressive* (*b* = 2.37, *p* < .001***) levels to be perceived as significantly more innovative compared to the *conventional*. In contrast, the overall *Perceived Innovativeness* in this model is significantly lower in *T2* (*b* = −0.31, *p* < .05*), again supporting our hypothesis that the perceived novelty in sound textures decreases with advancing elaboration. There are no other significant dynamic effects found in these models regarding the different levels of *Innovativeness*.

## Discussion

Our study focused on evaluating different acoustic concepts to determine a balanced degree of novelty in EV sound designs. In terms of the aesthetic preference for a suitable sound design of an EV's acceleration process, our results indicate that higher degrees of novelty are appreciated (dimension of *Liking*), as overall, the *progressive* and *futuristic* concepts in our study are liked significantly more than the less innovative concepts. The empirical data of *Perceived Innovativeness* can validate our conception of novelty content in the used stimuli: the *progressive* and *futuristic* levels are perceived as significantly more innovative. In contrast, the *technical* group of *Sound Character* is perceived as significantly less innovative and significantly liked less. As aesthetic preference and novelty perception are time-dependent variables and develop with increasing elaboration and exposure to a product, our study methodologically focused on dynamic effects in perception. This was achieved by applying repeated evaluation of the core dimensions through the *RET* (Carbon, 2015; Carbon & Leder, 2005; Faerber et al., 2010). Our findings demonstrate the *Perceived Innovativeness* to wear off over time with increasing elaboration (comparison of *T1* and *T2*) of the sound textures. For the specific factor levels, we did not find significant differences. Nonetheless, our graphs (Figures 6 and 8) indicate that this decreasing effect seems to be weaker for concepts with novel elements (*artistic* concepts) and more innovativeness (*progressive* and especially the *futuristic* level). These tendencies should be further investigated in future research. Regarding the aesthetic preference, our findings found *Liking* overall to increase with extended elaboration. Though our data shows no significant differences between the *Innovativeness Levels* or *Sound Character* groups, the graphical depictions (Figures 6 and 8) show slight tendencies that stimuli with higher novelty content (*artistic* and/or *futuristic* sound textures) have a steeper increase of *Liking* over time. This as well should be further investigated in future research.

Regarding the semantics conveyed by the sound textures used in this study, *technical* sound concepts are perceived as significantly less sustainable, pleasant, and emotional than *artistic* concepts. The data also shows this effect for the more innovative sound concepts: the more innovative stimuli, represented by the *futuristic* and *progressive* levels, are perceived as significantly more sustainable, pleasant, and emotional. These effects are even stronger for our study's *futuristic* level, comprising the highest degree of innovative content. In the context of EV sound design, these semantics (*Perceived Sustainability*, *Perceived Pleasantness*, *Perceived Emotionality*) should be considered closely, as such characteristics might be beneficial in creating a suitable sound design for such novel vehicle types in the future. For the semantic dimension of *Perceived Powerfulness* though, the more familiar concepts seem to be beneficial, as the stimuli from the *technical* concepts (*Sound Character*) are perceived as significantly more powerful. Although the correlation between the semantic dimensions between *Perceived Powerfulness* and *Perceived Sustainability* (*r* = −.61, *p* = .062) does not confirm a significant negative association, it indicates a clear negative trend. Whether this trend and the higher scores of more *technical* sound concepts in *Perceived Powerfulness* can be assumed as an artifact effect due to decades of associative learning or proves to be a time-stable finding is to be ascertained by future research. These as specifically powerful perceived sound characteristics, incorporated in the more familiar sound textures, might just be a temporary, but well-established link to the prevailing idea of a powerful vehicle. This should, therefore, be re-considered and reviewed when more people have had a chance to experience electrified driving more thoroughly and build upon their learning history. For the moment, our results indicate that *Perceived Powerfulness*, as we construe it from more ICEV-oriented sound concepts, is a killer for *Perceived Sustainability*.

Another important finding that should be considered for EV sound design in the context of the investigated semantic dimensions in this study is that some semantics are associated with each other, as for example, *Perceived Pleasantness* and *Perceived Sustainability*, or *Perceived Innovativeness* with *Perceived Sustainability*. Another important finding of our research is that aesthetic preference and novelty perception are significantly positively correlated, indicating innovativeness in the sound design of EVs to be appreciated. Interestingly, most associations between *Liking* and *Perceived Innovativeness* with the other semantic dimensions intensify with increasing elaboration. These findings emphasize that repeated exposure to novel sound material is needed to obtain more thorough evaluations and should be accounted for in future studies on aesthetic preference.

Future studies should also address the limitations of the present study. For example, more immersive experimental environments, such as simulators or real vehicles, could be considered in upcoming research, as in the current setup, we were limited to a listening lab. Moreover, further sound textures should be investigated in the future. The stimulus set was carefully considered but is limited to the 10 chosen sound textures. While the number of evaluated stimuli is suitable for an experiment with repeated evaluation considering cognitive capacities on the side of the participants, future studies should carefully consider their sound concepts for the evaluation. To preselect a final stimulus set, we recommend a representative sample to evaluate the sound material in a pre-study. In our case, the preassignment through experts worked well, but we see possible improvement in this aspect of our study. With a carefully chosen stimulus set for a final study, researchers can aim at a more balanced ratio among the levels of the variation factors and validate the preassigned categories. As a last point, our study showed dynamic effects in the appreciation of novelty in EV sound concepts over time. Therefore, the results of our study, just as well as any study investigating the preference for innovative sound material at a certain time, might be confounded with Zeitgeist effects, similar to the findings from Carbon (2010) regarding the appreciation of curvature in design. To consider such effects in EV sound concepts in future studies would be interesting to determine long-term trends in EV sound design.

## Conclusion

The findings of this study provide an initial orientation regarding the degree of innovativeness in sound textures that can be used for acoustic enhancement in electrified driving. Consistent with our hypothesis, our results indicate that sound concepts that are perceived as more innovative and characterized by high degrees of novelty and unprecedented sound elements seem to be liked less at first glance—or, in this case, audition—and rejected by customers. With increasing user experience and exposure to the novel EV soundscapes, such sound design approaches will be accepted and even increasingly appreciated and preferred. Our results suggest that more progressive, futuristic, and artistic sound concepts are more suitable for EVs that represent a novel era of transportation technology. Moreover, the importance of extended elaboration through repeated exposure when it comes to the evaluation of innovative designs with novel content is demonstrated in this study. Manufacturers are still left with the decision to direct their sound design strategies toward one of many possible approaches: to resemble familiar, well-established driving sound from the combustion engine's era or to dare innovative approaches, shaping novel vehicle soundscapes. This study, though, indicates that, especially in the context of electrified driving, high degrees of innovativeness and novel elements in sound concepts are appreciated in an EV's acoustic design. Through applied sound textures in the product's design, the vehicle's character can be diversified in manifold ways. Consistent with our hypotheses, the results of this study confirm that specific sound characteristics are associated with specific semantics: more pleasant perceived sounds are better liked, more innovative concepts are perceived as more sustainable. Here, the orchestration of actively enhanced acoustic feedback and passive soundscape design needs to be carefully considered, as the sound textures potentially influence the customers’ overall user experience, performance, and interaction with the product. As an outlook, we would like to encourage future studies to consider holistic development approaches in the acoustic design of EVs and to consider dynamic effects in novelty perception and aesthetic preference.

## Notes

1. We originally planned to perform standard GLMs to analyze the data and are aware that using G*Power is not the most suitable tool to perform power analyses for LMMs. We would like to stress that fixed effects in LMMs overlap with those in GLMs, and running a power analysis on the GLM gives at least a good approximation for how well the study is powered to detect main effects.

## References

[bibr1-20416695251323786] Allman-Ward M. Franks G. Di Nenno G. Miglietta P. (2020). A method for designing and delivering interior and exterior noise of an electric vehicle. In Siebenpfeiffer W. (Ed.), Proceedings of Automotive Acoustics Conference 2019 (pp. 47–63). Springer Fachmedien Wiesbaden. 10.1007/978-3-658-27669-0_4

[bibr2-20416695251323786] Allman-Ward M. Williams R. Heinz T. Demontis M. (2014). Designing and delivering the right sound for quiet vehicles. INTER-NOISE and NOISE-CON Congress and Conference Proceedings (pp. 4443–4451.2493).

[bibr3-20416695251323786] Baha E. Lu Y. Brombacher A. Mensvoort van K. (2012). Most advanced yet acceptable, but don`t forget. In DS 71: Proceedings of NordDesign (pp. 22–24). 9th NordDesign Conference.

[bibr4-20416695251323786] Blickensdorff J. Boulliung M. Burkard M. Dold C. Emretsson B.-G. Genuit K. Graf B. Kurch M. Millithaler P. Mohr C. Nichols M. Pecher A. Richter M. Rittgerott T. Satzinger S. Stretz D. Ulz A. (2019). Akustik. In Tschöke H. Gutzmer P. Pfund T. (Eds.), Elektrifizierung des antriebsstrangs (pp. 307–363). Springer. 10.1007/978-3-662-60356-7_20

[bibr5-20416695251323786] Bodden M. Belschner T. (2016). Principles of active sound design for electric vehicles.In INTER-NOISE and NOISE-CON Congress 2016 (pp. 1693–1697).

[bibr6-20416695251323786] Borg K. L. (2014). Introduction: Constructing sociotechnical environments—Aurality, air quality, and automobiles. Technology & Culture, 55, 287–298. 10.1353/tech.2014.0049

[bibr7-20416695251323786] Carbon C.-C. (2010). The cycle of preference: Long-term dynamics of aesthetic appreciation. Acta Psychologica, 134(2), 233–244. 10.1016/j.actpsy.2010.02.004 20236624

[bibr8-20416695251323786] Carbon C.-C. (2015). Predicting preferences for innovative design: The “repeated evaluation technique” (RET). NIM Marketing Intelligence Review, 7, 34–39. 10.1515/gfkmir-2015-0016

[bibr9-20416695251323786] Carbon C.-C. Leder H. (2005). The repeated evaluation technique (RET): A method to capture dynamic effects of innovativeness and attractiveness. Applied Cognitive Psychology, 19, 587–601. 10.1002/acp.1098

[bibr10-20416695251323786] Carson S. J. Jewell R. D. Joiner C. (2007). Prototypicality advantages for pioneers over me-too brands: The role of evolving product designs. Journal of the Academy of Marketing Science, 35, 172–183. 10.1007/s11747-007-0043-3

[bibr11-20416695251323786] Cerrato G. (2009). Automotive sound quality—Powertrain, road and wind noise. Sound & Vibration, 43, 16–24.

[bibr12-20416695251323786] Clendinning E. A. (2018). Driving future sounds: Imagination, identity and safety in electric vehicle noise design. Sound Studies, 4, 61–76. 10.1080/20551940.2018.1467664

[bibr13-20416695251323786] Cohen J. (1988). Statistical Power Analysis for the Behavioral Sciences. Erlbaum.

[bibr14-20416695251323786] Devillers E. Gning P. Degrendele K. Le Besnerais J. (2020). Sound quality aspects of electric vehicles. ATZ Worldwide, 122, 26–31. 10.1007/s38311-020-0254-5

[bibr15-20416695251323786] Eisele G. Kauth M. Steffens C. Glusk P. (2019). Automotive megatrends and their impact on NVH. In Bargende M. Reuss H.-C. Wagner A. Wiedemann J. (Eds.), Proceedings of 19th Internationales Stuttgarter Symposium (pp. 523–539). Springer Fachmedien Wiesbaden. 10.1007/978-3-658-25939-6_45

[bibr16-20416695251323786] Faerber S. J. Leder H. Gerger G. Carbon C.-C. (2010). Priming semantic concepts affects the dynamics of aesthetic appreciation. Acta Psychologica, 135, 191–200. 10.1016/j.actpsy.2010.06.006 20615491

[bibr17-20416695251323786] Faul F. Erdfelder E. Lang A.-G. Buchner A. (2007). G*Power 3: A flexible statistical power analysis program for the social, behavioral, and biomedical sciences. Behavior Research Methods, 39, 175–191. 10.3758/BF03193146 17695343

[bibr18-20416695251323786] Fiebig A. Schulte-Fortkamp B. (2019). Acceptance of synthetic driving sounds in the interior of electric vehicles. In Siebenpfeiffer W. (Ed.), Proceedings of Automotive Acoustics Conference 2015 (pp. 18–34). Springer Fachmedien Wiesbaden. 10.1007/978-3-658-27648-5_2

[bibr19-20416695251323786] Franke T. Attig C. Wessel D. (2019). A personal resource for technology interaction: Development and validation of the affinity for technology interaction (ATI) scale. International Journal of Human–Computer Interaction, 35, 456–467. 10.1080/10447318.2018.1456150

[bibr20-20416695251323786] Gavric L. (2020). NVH refinement issues for BEV. In Siebenpfeiffer W. (Ed.), Proceedings of automotive acoustics conference 2019 (pp. 1–9). Springer Fachmedien Wiesbaden. 10.1007/978-3-658-27669-0_1

[bibr21-20416695251323786] Genuit K. (2012). (R)Evolution in vehicle acoustics—Sound design, warning signals and quiet cities. The Journal of the Acoustical Society of America, 131, 3301. 10.1121/1.4708343

[bibr22-20416695251323786] Genuit K. Fiebig A. (2014). Sound design of electric vehicles—challenges and risks. In INTER-NOISE and NOISE-CON Congress and Conference Proceedings (pp. 3492–3501).

[bibr23-20416695251323786] Heiskanen E. Hyvönen K. Niva M. Pantzar M. Timonen P. Varjonen J. (2007). User involvement in radical innovation: Are consumers conservative? European Journal of Innovation Management, 10, 489–509. 10.1108/14601060710828790

[bibr24-20416695251323786] Hekkert P. Snelders D. van Wieringen P. C. W. (2003). “Most advanced, yet acceptable”: Typicality and novelty as joint predictors of aesthetic preference in industrial design. British Journal of Psychology, 94, 111–124. 10.1348/000712603762842147 12648393

[bibr25-20416695251323786] Karlsson B. Aronsson N. Svensson K. (2003). Using semantic environment description as a tool to evaluate car interiors. Ergonomics, 46, 1408–1422. 10.1080/00140130310001624905 14612328

[bibr26-20416695251323786] Kleinjohann M. (2020). Einsatzfelder des acoustic branding. In Kleinjohann M. (Ed.), Essentials. Marketingkommunikation mit acoustic branding (pp. 19–45). Springer Fachmedien Wiesbaden. 10.1007/978-3-658-29989-7_4

[bibr27-20416695251323786] Krishna G. (2021). Understanding and identifying barriers to electric vehicle adoption through thematic analysis. Transportation Research Interdisciplinary Perspectives, 10, 100364. 10.1016/j.trip.2021.100364

[bibr28-20416695251323786] Leder H. Carbon C.-C. (2005). Dimensions in appreciation of car interior design. Applied Cognitive Psychology, 19, 603–618. 10.1002/acp.1088

[bibr29-20416695251323786] Lennström D. Argen A. Nykänen A. (2011). Sound quality evaluation of electric cars—preferences and influence of the test environment. In Aachener Acoustic Colloquium (pp. 95–100).

[bibr30-20416695251323786] Loewy R. (1951). Never leave well enough alone: The personal record of an industrial designer from Lipsticks to locomotives. Simon & Schuster.

[bibr31-20416695251323786] Ma C. Chen C. Liu Q. Gao H. Li Q. Gao H. Shen Y. (2017). Sound quality evaluation of the interior noise of pure electric vehicle based on neural network model. IEEE Transactions on Industrial Electronics, 64, 9442–9450. 10.1109/TIE.2017.2711554

[bibr32-20416695251323786] Münder M. Carbon C.-C. (2022a). Howl, whirr, and whistle: The perception of electric powertrain noise and its importance for perceived quality in electrified vehicles. Applied Acoustics, 185, 108412. 10.1016/j.apacoust.2021.108412

[bibr33-20416695251323786] Münder M. Carbon C.-C. (2022b). A literature review [2000–2022] on vehicle acoustics: Investigations on perceptual parameters of interior soundscapes in electrified vehicles. Frontiers in Mechanical Engineering, 8, Article 974464. 10.3389/fmech.2022.974464

[bibr34-20416695251323786] Noble C. H. Kumar M. (2008). Using product design strategically to create deeper consumer connections. Business Horizons, 51, 441–450. 10.1016/j.bushor.2008.03.006

[bibr35-20416695251323786] Ortlieb S. A. Kügel W. A. Carbon C.-C. (2020). Fechner (1866): The aesthetic association principle—A commented translation. i-Perception, 11, 2041669520920309. 10.1177/2041669520920309 32528640 PMC7264472

[bibr36-20416695251323786] Qatu M. S. (2012). Recent research on vehicle noise and vibration. International Journal of Vehicle Noise and Vibration, 8, 289–301. 10.1504/IJVNV.2012.051536

[bibr37-20416695251323786] Qatu M. S. Abdelhamid M. K. Pang J. Sheng G. (2009). Overview of automotive noise and vibration. International Journal of Vehicle Noise and Vibration, 5, 1–35. 10.1504/IJVNV.2009.029187

[bibr38-20416695251323786] Rauthmann J. F. (2017). Persönlichkeitspsychologie: Paradigmen—Strömungen—Theorien. Springer. 10.1007/978-3-662-53004-7

[bibr39-20416695251323786] Sinambari G. R. Sentpali S. (2020). Ingenieurakustik. Springer Fachmedien Wiesbaden. 10.1007/978-3-658-27289-0

[bibr40-20416695251323786] Soto C. J. John O. P. (2017). The next Big Five Inventory (BFI-2): Developing and assessing a hierarchical model with 15 facets to enhance bandwidth, fidelity, and predictive power. Journal of Personality and Social Psychology, 113, 117–143. 10.1037/pspp0000096 27055049

[bibr41-20416695251323786] Sottek R. Krebber W. Stanley G. R. (2005). Tools and methods for product sound design of vehicles. *SAE Technical Paper Series*, Article 2005-01-2513. 10.4271/2005-01-2513

[bibr42-20416695251323786] Streicher B. Lange S. Eisele G. Steffens C. (2021). Effiziente methoden im Active Sound Design. ATZ Automobiltechnische Zeitschrift, 123, 58–61. 10.1007/s35148-020-0623-9

[bibr43-20416695251323786] Swart D. J. Bekker A. (2019). The relationship between consumer satisfaction and psychoacoustics of electric vehicle signature sound. Applied Acoustics, 145, 167–175. 10.1016/j.apacoust.2018.09.019

[bibr44-20416695251323786] Swart D. J. Bekker A. Bienert J. (2018). The subjective dimensions of sound quality of standard production electric vehicles. Applied Acoustics, 129, 354–364. 10.1016/j.apacoust.2017.08.012

[bibr45-20416695251323786] Zeller P. (2018). Handbuch Fahrzeugakustik. Springer Fachmedien Wiesbaden. 10.1007/978-3-658-18520-6

